# MIGRATION AS A PATHWAY TO KINLESSNESS AMONG OLDER ADULTS LEFT BEHIND

**DOI:** 10.1093/geroni/igad104.1839

**Published:** 2023-12-21

**Authors:** Amilcar Matos-Moreno, Alexis Santos-Lozada, Mara Sheftel, Ashton Verdery

**Affiliations:** Pennsylvania State University, State College, Pennsylvania, United States; Pennsylvania State University, State College, Pennsylvania, United States; Penn State University, State College, Pennsylvania, United States; The Pennsylvania State University, University Park, Pennsylvania, United States

## Abstract

Recent research examines the topic of kinless adults, people over the age of 50 who have neither a living spouse nor living children. Crossnational examinations find that the prevalence of kinlessness in these age groups ranges between 2% and 10% in most countries around the world and that kinless individuals tend to be disadvantaged on health and well-being dimensions. Prior work has not, however, examined migration in the context of kinlessness. Given high rates of migration in numerous sending contexts around the world, this study examines the prevalence of kinlessness in Puerto Rico, Mexico, Costa Rica, and the United States incorporating migration as a pathway to kinlessness and examining its association with well-being. Using surveys from the Health and Retirement family of studies from Puerto Rico, Mexico, Costa Rica, and the United States, we estimate the prevalence of kinlessness focusing on how these estimates change when treating adult family migrants as not available for the provision of social support. We also estimate the association between kinless typologies and health status. Puerto Rico exhibits the highest older adult kinlessness prevalence (Puerto Rico: 8.4%, Costa Rica:6.0%, Mexico:6.5%, United States:7.2%). The incorporation of migration as a pathway to kinlessness increases the prevalence in Puerto Rico (+3.8%) and Mexico (+5.2%) more so than for Costa Rica or United States. Kinlessness is associated with higher levels of depression among Puerto Rican older adults. This study reveals the importance of considering migration as a pathway to kinlessness in high outmigration countries and its impact on health.

